# First host record of stylopization of a worker ant, *Ectatomma edentatum* (Formicidae: Ectatomminae), by a Myrmecolacidae (Strepsiptera)

**DOI:** 10.3389/finsc.2026.1762540

**Published:** 2026-02-05

**Authors:** André L. Marambaia, Jacques H. C. Delabie, Favízia F. de Oliveira, Jeyaraney Kathirithamby

**Affiliations:** 1 Laboratório de Bionomia, Biogeografia e Sistemática de Insetos (BIOSIS), Instituto de Biologia (IBIO), Universidade Federal da Bahia (UFBA), Salvador, BA, Brazil; 2 Laboratório de Mirmecologia, Centro de Pesquisas do Cacau, Comissão Executiva do Plano da Lavoura Cacaueira (CEPLAC), Universidade Federal da Bahia & Universidade Estadual de Santa Cruz, Ilheus, BA, Brazil; 3 Department of Biology, University of Oxford, Oxford, United Kingdom

**Keywords:** cephalotheca, *Ectatomma*, Hymenoptera, Myrmecolacidae, new record, Strepsiptera, stylopization

## Abstract

Strepsipterans of the family Myrmecolacidae are endoparasitoid insects of ants distributed worldwide, except in the Palearctic and Antarctic regions. Despite this, knowledge about their host ants and the effects of this parasitism on their biology remains scarce. To fill this gap in Brazil, we used yellow pan traps in a fragment of Atlantic Forest in search of stylopized ants (i.e., parasitized by Strepsiptera). The present study records for the first time the observation of a stylopized worker ant of the species *Ectatomma edentatum* in Brazil and details the general behavior of stylopized ants. Furthermore, we discuss the potential of yellow pan traps as a collection method of stylopized ants and compare their functionality to previously employed methods.

## Introduction

Strepsiptera are entomophagous endoparasites that stylopize seven orders of Insecta and is one of the least species-rich taxa among insect parasitoids. This order consists of 14 families, and their known hosts are distributed across 36 families in seven orders of Insecta (1, 2). The Strepsiptera biodiversity recorded include 603 species of Strepsiptera described worldwide (1, 2). In Brazil, 33 Strepsiptera have been recorded so far (3); however, many more remain undescribed. The strepsipteran family Myrmecolacidae Saunders, 1872, exhibits sexually dimorphic host relationships known as heterotrophic heteronomy, where males stylopize Formicidae Latreille, 1802 (ants), and females stylopize Orthoptera (crickets, grasshoppers) and Mantodea (mantids) (1, 2, 4–6). Heterotrophic heteronomy [*sensu* Walter (7)] is an unusual form of polymorphism and is rare not only in Strepsiptera but also in insect parasitoids in general. This phenomenon occurs convergently in two lineages of sexually dimorphic parasitic insects: the Myrmecolacidae (Strepsiptera) and the Aphelinidae Thomson, 1876 (Hymenoptera, Chalcidoidea).

Males are matched to conspecific females by molecular characterization, and so far, only two species have been unequivocally matched (5, 6). Majority of the Myrmecolacidae described (approximately 93 so far) consist of free-living males that have been found in traps. However, only hosts of 23 species (from nine genera of six subfamilies) stylopize ant hosts (2), which is approximately a quarter of the total male Myrmecolacidae described. This is curious as ants comprise the largest group of invertebrates in any habitat, yet stylopized ants are seldom encountered (8).

Looking for stylopization by males of Myrmecolacidae, the entire contents of ant nests have been examined; however, these attempts have been largely unsuccessful Kathirithamby personal observation. In addition, Ogloblin (9), then Kathirithamby et al. (6) and Nakase et al. (10), observed changes in the behavior of positive phototropism and elevation in stylopized ants, which are hypothesized to be mediated by parasites (11).

It is speculated that, when ants are stylopized, they remain in the nest even after the extrusion of the male cephalotheca, while the male strepsipteran undergoes pupation (within the nest) (2). The cephalotheca of the male strepsipteran is the cap of the puparium that extrudes from the host. It generally has the same coloration as the ant’s cuticle, which may act as a camouflage (2). As far as is known, stylopized ants in the nests are not attacked by mates; however, if they were to be active outside the nest, they would be prone to predation (8). Just before the male myrmecolacid emerges, the ant leaves the nest, climbs to a high point on vegetation, and, at this vantage point, the free-living male strepsipteran emerges from the puparium. After this, the ant with its empty puparium, which is susceptible to a fungal infection, dies (Kathirithamby, personal observation).

The ants of the genus *Ectatomma* Smith, 1858 (Formicidae: Ectatomminae), are opportunistic omnivores, primarily functioning as generalist predators. They inhabit subterraneous nests with a medium population density (~50–200 inhabitants) and forage on the ground (12). Here, we document a new host record of the stylopization of a worker of the ant *Ectatomma edentatum* Roger, 1893, by male Myrmecolacidae (Strepsiptera).

## Materials and methods

Sampling was conducted in an urban fragment of Atlantic Forest in the city of Salvador, Bahia State, in the northeast region of Brazil (12°57′ S, 38°26′ W). Specimens were collected using 16 yellow pan traps (dishes with a maximum diameter of 21.5 cm, an internal diameter of 17 cm, and a depth of 3 cm) at ground level with a distance between them of 30–50 cm, between October 8 and 12, 2025. The trays were filled with 10%–20% aqueous solution of commercial NaCl and 1 mL of commercial neutral detergent per liter solution. A Leica MZ125 stereomicroscope with an attached Leica Flexacam C5 camera (Leica Microsystems, Wetzlar, Germany) was used to capture images of the specimens. The illustration was created using Adobe Illustrator 2025 software. Host and parasitoid identification was performed based on the identification keys (13–15) for Neotropical entomofauna and with the assistance of specialists (J.H.C. Delabie and J. Kathirithamby, respectively). The specimen obtained was deposited in the entomological collection of the Natural History Museum of the Federal University of Bahia (MHNBA-UFBA, Salvador, Brazil).

## Results

One stylopized worker specimen of the ant *E*. *edentatum* (Formicidae: Ectatomminae) was collected (ID no. MZUFBA 00461).

### Description of the partially detached hinged cephalotheca of the Myrmecolacidae

Examined material: 1♂ Stylopized cephalotheca cap in the host, “Beco da Coruja” Street, Saboeiro neighborhood, Atlantic Forest fragment, City of Salvador, State of Bahia, Brazil (12°57′ S, 38°26′ W). 08-12. X. 2025. Yellow pan trap. A. L. Marambaia *Leg*. (ID no. MZUFBA Strepsip. 00461).

Measurements: Head: length, 492 µm; width, 760 µm. Compound eye: length, 237 µm; width, 179 µm. Distance between compound eyes, 363 µm. Antenna rudiment: external diameter, 74 µm; internal diameter, 30 µm. Distance between the base of the mandible and the compound eye, 70 µm; mandible length, 71 µm; distance between the apex of the mandibles, 178 µm; maxilla length, 57 µm; distance between the apex of the maxillae, 212 µm. Clypeus: length, 98 µm; width, 86 µm. Oral opening: length, 49 µm; width, 92 µm.

**Figure 1:**
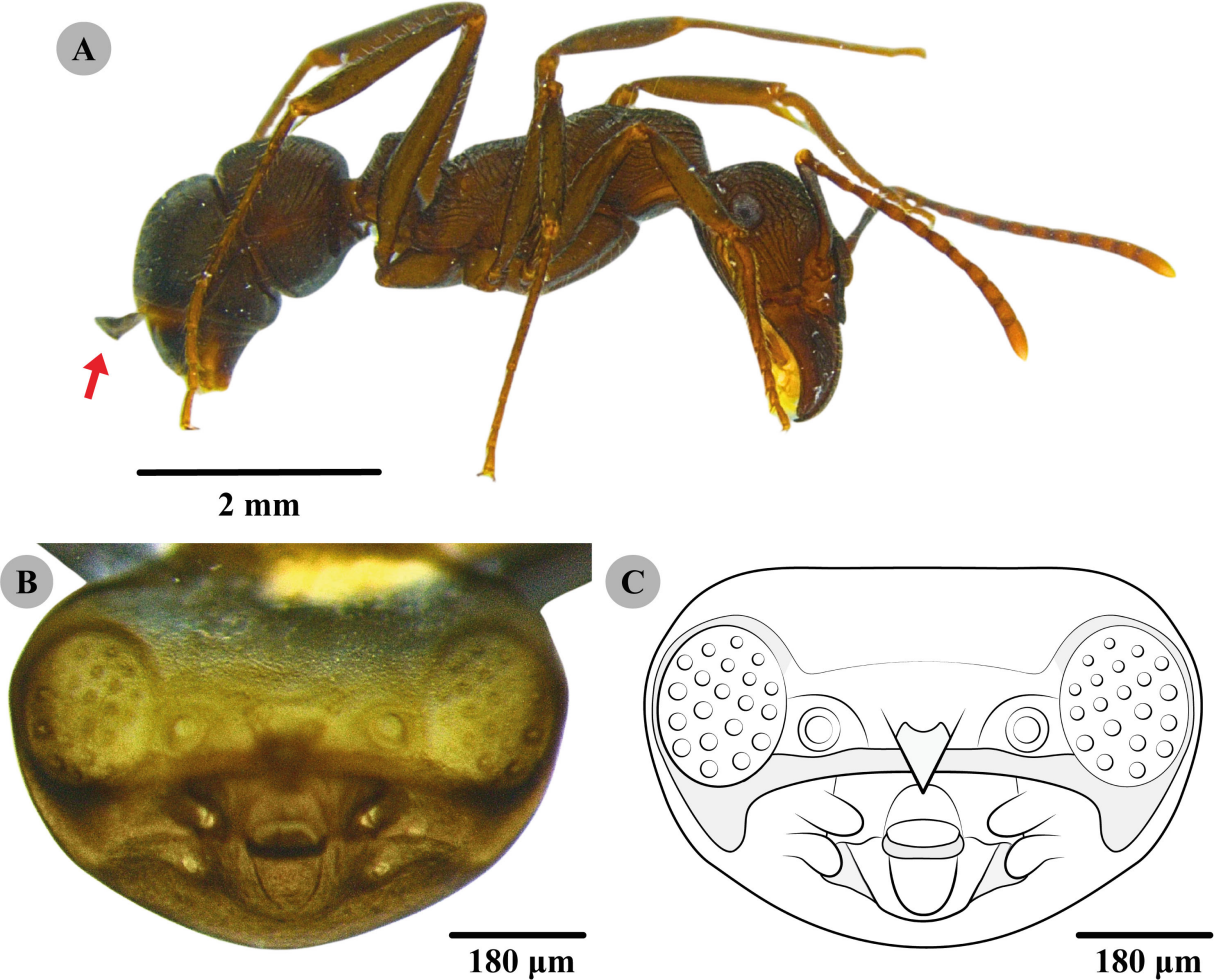
**(A)** Worker ant, *Ectatomma edentatum* Roger, 1893 (Formicidae: Ectatomminae), with a hinged cephalotheca (*arrow*). **(B)** Cephalotheca of Myrmecolacidae (Insecta: Strepsiptera). **(C)** Drawing of the cephalotheca of Myrmecolacidae (Insecta: Strepsiptera). Images and illustration produced by the author (A.L. Marambaia).

Description: (**Figures 1A–C**) The head (=hinged cephalotheca) is well sclerotized and brownish, with a semi-elliptical shape, and is distinctly wider than long. Compound eyes are well developed, large (1.3 times longer than wide), and with approximately 20 rudimentary ommatidia. Below the compound eyes, the head presents a more sclerotized and darkened transverse line. There is a small antennal rudiment with an external diameter about one-fifth of the distance between the compound eyes. There is an elongated clypeus with a subtriangular shape. Mandibles and maxillae are present, but are poorly developed and without teeth. The distance between the base of the mandible and the compound eye has the same length as the mandible. The oral opening is conspicuous and sub-elliptical, measuring approximately twice as wide as it is long. Head in lateral view, notably protruding in its medial region.

Remarks: The cephalotheca of the species of Myrmecolacidae described here is relatively similar to that of *Myrmecolax incautus* Oliveira & Kogan, 1959, described by Kathirithamby et al. (7) However, it presents smaller antennal rudiments (particularly in external diameter), measuring one-quarter of the distance between the compound eyes, which, when compared with the larger size of the rudiments in *M*. *incautus*, measure one-third of the distance between the compound eyes. The observed specimen presents less sclerotization in the medial transverse line, while this region is more sclerotized in *M*. *incautus*. Another difference is that the cephalotheca cap described in this study has approximately 20 ommatidia rudiments compared with the 30 ommatidia present in the description of *M*. *incautus*.

Comparing the cephalotheca of Myrmecolacidae species described by Nakase et al. (10), the shape of the specimen described here is well developed and subtriangular, while the one described by Nakase et al. (10) is smaller and rounded. The smaller diameter of the antennal rudiments in the specimen occupies approximately one-quarter of the distance between the compound eyes, whereas in the specimen described by Nakase et al. (10), it occupies one-third of the distance between the compound eyes. Furthermore, there is greater sclerotization in the transverse line superior to the compound eyes, a feature not seen in our specimen. In addition, the compound eyes described by Nakase et al. (10) appear proportionally smaller (similar length and width) compared with those described in this study, which are longer than wide. The base of the mandible of the specimen of Nakase et al. (10) is close to the compound eyes, whereas in our specimen, the base of the mandible is at least as far from the compound eyes as the length of the mandible. The maxilla is underdeveloped in the specimen described by Nakase et al. (10), in contrast to the maxillae described here, which are small, conspicuous, and almost as long as the mandibles.

The Myrmecolacidae parasitized in the ant discussed above cannot be identified to the species level as the male myrmecolacid had emerged from the host. Currently, identification of the cephalothecae of the male Myrmecolacidae to the species level is only possible by having a free-living male for comparison since only a few cephalothecae are known.

## Discussion

The ant that was collected contained an empty male puparium with a hinged cephalotheca (**Figures 1A–C**), which represents the first host record of stylopization of the species *E. edentatum* (Formicidae: Ectatomminae). Although the free-living male strepsipteran had emerged, the cap of the puparium, the cephalotheca, remained hinged to the host ant.

Stylopized ants with a hinged cephalotheca have also been reported for *Pheidole* sp. (Myrmicinae) stylopized by the male puparium of *Caenocholax* sp. (Strepsiptera: Myrmecolacidae) from Tapachula, Chiapas, Mexico (15); a minor worker of *Pheidole* sp. (Myrmicinae) stylopized by the male puparium of *Stichotrema robertsoni* Kathirithamby, 1991 (16) (Strepsiptera: Myrmecolacidae), from Natal, South Africa; and an alate *Myrmelachista zeledoni* Emery, 1896 (Formicinae), stylopized by the male puparium of *Caenocholax* sp. from Puntarenas Province, Wilson Botanic Gardens, Costa Rica (17). The reason for the hinged cephalothecae remains unknown.

Emerging strepsipteran males have been observed breaking the cap at the “line of weakness” [*sensu* Kathirithamby et al. (18)] of the puparium of *Elenchus tenuicornis* (Kirby, 1815) (Strepsiptera: Elenchidae) with an analogous structure, similar to the ptilinum of Diptera (19), or by employing their mandibles, as seen in *Xenos peckii* Kirby, 1813 (Strepsiptera: Xenidae) (20). In both instances, the cephalotheca completely detaches from the puparium, along its “line of weakness.” The mechanism the male Myrmecolacidae uses to “cut” along the “line of weakness” remains unknown; however, this line might not be circular, as in other Strepsiptera, which could allow the cap to remain hinged to the ant host after the emergence of the male strepsipteran.

Finding a stylopized ant in a yellow pan trap is a result that deserves attention. As previously reported, stylopized ants do not usually leave the nests until the free-living males are ready to emerge from their cephalothecae (1, 2). This makes their collection difficult through passive sampling and makes our result unprecedented for sampling stylopized ants since their collection commonly occurs through active field collection or captive rearing (1, 2, 6, 10) methods with higher costs and sampling effort. Considering this information, we propose two possible hypotheses to explain our unprecedented results. In the first hypothesis, the ant would have accidentally fallen from the vegetation above into the traps after dying from fungal infection, as this type of infection tends to kill the hosts relatively quickly after the male emerges (1, 2). However, the time period in which the ant is infected by fungi resulting in its death is still unknown. Thus, a second hypothesis for our results would be that the stylopized ant was attracted to the yellow pan trap before the infection became fatal to the ant. This latter hypothesis is corroborated by the fact that non-stylopized ants of the species *E*. *edentatum* are frequently collected in these traps (André L. Marambaia, personal observation). This shows that, even though they are terrestrial, these ants tend to be attracted to this type of trap, which can also occur with stylopized specimens.

Yellow pan traps should be considered as a collection method for Strepsiptera as they constitute a passive and low-cost method that can provide valuable results for understanding the behavior of stylopized ants, as well as the biology and the distribution of Myrmecolacidae. Yellow pan traps can therefore expand the possibility of obtaining stylopized ants in conjunction with active searching and captive rearing (6, 10), providing more data, such as those presented here. In addition, we can suggest that the combination of these methods with pitfall traps, Winkler extractors, and soil sampling tends to expand the opportunities for collecting stylopized ants since they are quite efficient in capturing ant diversity and abundance (21, 22).

This study reveals the first record of stylopization of the ant *E*. *edentatum* in yellow pan traps. As the male of the Myrmecolacidae family had already emerged from the host ant, no molecular study was performed. However, future collections that obtain ants with prior stylopization justify conducting such studies. The study of museum material containing ants with prior stylopization is also a useful and recommended exercise. Finally, the use of yellow pan traps for collecting stylopized ants, such as those used in this study, should be considered in conjunction with other commonly used collection methods to expand collection opportunities due to its low cost and reduced sampling effort.

## Data Availability

The raw data supporting the conclusions of this article will be made available by the authors, without undue reservation.
